# Deficits in visuo-spatial but not in topographical memory during pregnancy and the postpartum state in an expert military pilot: a case report

**DOI:** 10.1186/1756-0500-7-524

**Published:** 2014-08-13

**Authors:** Laura Piccardi, Paola Verde, Filippo Bianchini, Fabio Morgagni, Cecilia Guariglia, Felice Strollo, Enrico Tomao

**Affiliations:** Life, Health and Environmental Science Department, University of L’Aquila, L’Aquila, Italy; Neuropsychology Unit, IRCCS Fondazione Santa Lucia, Rome, Italy; Centro Sperimentale Volo – Reparto Medicina Aeronautica e Spaziale, Aeroporto “M. de Bernardi” Pratica di Mare Pomezia, Rome, Italy; Dipartmento di Psicologia, Sapienza Università di Roma, Rome, Italy; UOC Endocrinologia e Malattie Metaboliche, INRCA- IRCCS Roma, Rome, Italy; Corpo Sanitario Aeronautico, Rome, Italy

**Keywords:** Spatial cognition, Pregnancy, Postpartum, Human navigation, Sex differences

## Abstract

**Background:**

It is well known that cognitive and emotional changes occur during pregnancy, but little is known about their magnitude or their time of occurrence and recovery. During pregnancy memory is one of the most impaired cognitive functions. Although long-term aspects of memory have been investigated, other aspects of memory have not yet been explored (i.e., navigational memory and reaching memory).

**Case presentation:**

Here we describe the changes in reaching and walking memory that occurred during pregnancy and one year after delivery in an Italian female military pilot (Case 1) with high spatial ability. In memory tests she showed a classical dissociation between performance in reaching and walking distance, which indicated a failure of working memory, learning, and storage in reaching space. This suggests that her expertise served as a protective factor mitigating her low walking memory performance, and saving the topographical component.

We compared her performance with that of two non-pregnant control groups (i.e., women pilots and non-pilots) and found that Case 1’s reaching memory performance was significantly worse than that of the control groups. Even one year postpartum, Case 1’s performance was not yet the same as that of the other pilots.

**Conclusions:**

These findings contribute to our knowledge of the specific, as yet unexplored, aspects of memory deficits in women pilots during pregnancy and postpartum and suggest the need for better neuropsychological assessment before these women return to work in operational environments.

## Background

Recently, a great deal of attention has been paid to cognitive changes during pregnancy and postpartum. Previously, Poser *et al.* [1] observed that over 80% of pregnant women reported increased forgetfulness. In a review of the literature on this topic, Brett and Baxendale [2] concluded that both forgetfulness and poor memory were top on the list of difficulties mentioned by pregnant women. In fact, several studies found that retrospective memory was adversely affected by pregnancy (e.g., see [2–4]) but other studies did not (see [5–7], and for a review, see [8]). Until now, studies of the memory changes that occur at different stages of pregnancy and at different times postpartum have investigated aspects of memory which vary with regard to temporal duration (working memory and long-term memory) and storage capacity. However, no deficits have been reported between pregnant and postpartum women regarding the storage component of working memory [9, 10]. Some studies found that pregnancy selectively affected the retrieval component of long-term memory, specifically in free recall [3, 4, 11]. Explicit and implicit memory have also been explored. Explicit memory requires the deliberate recall of information from a specific learning episode and implicit memory refers to the unconscious accessing of previous experiences in the absence of intentional recollection. Sharp *et al.* [12] found that irrespective of gravida status or trimester of pregnancy, pregnant women were significantly impaired on measures of both types of memory. More recently, however, McDowall and Moriarty [7] failed to identify any significant differences between pregnant and non-pregnant women on measures of implicit and explicit memory. In Henry and Rendell ‘s [8] meta-analytic study of memory changes in pregnancy it emerged that deficits were present only in tasks with high demands on effortful processing and, specifically, with measures of free recall and tasks involving the executive component of working memory. Henry and Rendell [8] also observed that the pattern of deficits observed postpartum mirrors the pattern of deficits observed during pregnancy both qualitatively and quantitatively. Furthermore, they found that women accurately estimated the quality of their performance; in fact, the effect sizes for self-reported memory deficits were similar in magnitude to the effect sizes observed for the behavioural measures [8]. Note that the magnitude of the deficits was relatively subtle, which might partially explain why different studies failed to find homogeneous results. Indeed, the results might depend on the sensitivity of the instrument used to detect the memory deficits. To our knowledge, no findings have been reported about memory in reaching and walking distance during pregnancy and postpartum.

The space around us is a multifactorial construct of our brain and distinct areas are responsible for coding space that is behaviourally defined as outside reaching distance (far space) and as within reaching distance (near space) (e.g., [13]). The coding of space as *near* and *far* is not only determined by arm-reaching distance, but also depends on how the brain represents the extension of the body space. There is an evolutionary reason for this distinction. Near space, also called reaching space, refers to the portion of space within “grasping distance” (i.e. the space in which a seated individual can grasp an object), whereas far space, also called navigational space [14], extends beyond our reach and has been called the space within “walking distance” [15]. Our brain distinguishes between far and near space because the relevance of actions performed by others may be different for the observer according to the region of space in which they are executed and can lead to different behavioural responses. Recently, it was demonstrated that the dissociation between reaching and walking space might not be limited to perception and action but might extend to memory systems, as also shown by functional magnetic resonance imaging (fMRI) [16–19]. In particular, the calcarine cortex, lingual gyrus and dorsolateral prefrontal cortex on the right side of the brain were selectively involved in learning within navigational space, and the middle occipital gyrus, inferior temporal gyrus and lingual and fusiform gyrus on the left side of the brain were selectively involved in learning within reaching space [19]. Visuo-spatial memory allows us to remember where objects around us are and provides an overview map of space that can be used for navigation. Reaching and walking space can be distinguished in visuo-spatial memory. In fact, there is increasing evidence that remembering items within grasping distance is different from remembering a pathway within walking distance. Recent studies of brain-damaged patients suffering from navigational deficits showed that they selectively failed in topographical memory tasks but not in reaching memory tasks [16–18]. Visuo-spatial memory is a multidimensional concept [20–22]. Indeed, It is relevant in brain-damaged patients who show dissociated performances and in distinct patterns due to individual differences (i.e., gender, age, spatial styles and differences in expertise). In general, memory performance can be influenced by several external and internal factors and people often report perceived memory deficits that do not necessarily reflect objective changes [8, 23].

In this study we subjected a female Italian Air Force pilot (Case 1) to reaching and walking distance memory tests and a hormonal assessment in the second trimester of pregnancy and one year postpartum. Previously, we investigated the capability of Case 1, during pregnancy and one year postpartum, to mentally rotate an object finding the presence of deficits [24]. Mental rotation is a basic skill for a pilot and for solving visuo-spatial memory tests, it is always assessed during the selection for entering in the Air Force Academy and all pilots demonstrate high mental rotation abilities. This first investigation suggests us to scrutinize other cognitive processes related to mental rotation and visuo-spatial abilities. To this purpose, in the present paper we assessed the ability of Case 1 in reaching distance memory (this ability in Aviation is used in managing on-line information coming from the instrument panel) and walking distance memory (a memory for navigational information coming from the external environment), both this types of memory are basic in successful flying. To measure reaching distance memory we used the Corsi Block-Tapping test (CBT; [25]) and to assess walking distance memory, the Walking Corsi Test (WalCT; [16, 26]). The latter test is a validated navigational variant of the CBT, which requires observing and reproducing spatial sequences by walking in a room. We investigated Case 1’s performance with regard to temporal duration (working memory and long-term memory) in both the CBT and the WalCT.

During her second trimester of pregnancy she showed a large deficit in the CBT but not in the WalCT. She was aware of the deficit, which almost completely disappeared one year postpartum. By studying Case 1 we were able to gain more knowledge about the memory changes that occur during pregnancy also in women with high-spatial ability and no complaints of depression, sleep disruption or fatigue. The dissociation between her performance in the reaching and walking distance memory tests is noteworthy because her expertise should have mitigated her low walking space memory performance. This effect can be explained as a protective factor resulting from her experience in human navigation, which prevented her complete visuo-spatial memory failure by saving the topographical component.

## Case presentation

Case 1 is a 32-year-old Italian female pilot of the Italian Air Force (ItAF). At the time of the study she had 680 hours of flight experience. She was assessed with reaching and walking distance memory tests twice in the second trimester of pregnancy and one year after delivery. Circulating levels of estradiol, progesterone and testosterone were measured on three different occasions during the second trimester of pregnancy and one year after delivery in the follicular phase of three different menstrual cycles. Estradiol levels were significantly different (p < .05), that is 798 ± 126 pMol/L and 181 ± 121 pMol/L. Progesterone was 151.6 ± 18.2 nMol/L during the second trimester and dropped to 1.9 ± 0.5 nMol/L (p < .05) during the follicular phase of the normal menstrual cycle. Finally, testosterone remained almost stable throughout the entire observation period: 2.2 ± 0.6 nMol/L during pregnancy vs. 2.9 ± 0.3 nMol/L postpartum (p = n.s.).

Her performance on the memory tests was compared with the performances of 10 women ItAF pilots (mean age 28.9 ± 2.8 years; mean education 18 ± 0) and with the validated normative data reported for the WalCT and CBT in Piccardi *et al.* [26]. Both groups (pilots and normative sample) were matched with Case 1 for age, gender and education.

The study was approved by the local ethical committee of the Experimental Flight Center, Aerospace Medicine Department, “M. de Bernardi ” Air Base (prot. n. 2012/09/24 RMAS), Italy. Written informed consent to participate in to the study was provided by Case 1 and by the control groups (both pilots and normative sample).

### Reaching distance memory

The CBT [25] is a widely used visuo-spatial memory task in which nine blocks (4.5 × 4.5 cm) are fixed on a baseboard (30 × 25 cm) in a scattered array. It tests both working memory and long-term memory. To test working memory (WM), the examiner taps a number of blocks at a rate of one block per 2 s, after which the subject has to tap the block sequence in the same order. The block sequences gradually increase in length (starting from a 2-block sequence); the score is the number of blocks in the longest sequence remembered correctly (block span).

We assessed two aspects of visuo-spatial long-term memory: learning (L) and delayed recall (DR). In the L part of the test, she had to learn an eight-block sequence (following the procedure described in [16, 26]) demonstrated by the examiner. The learning criterion was reached if she reproduced the correct sequence three times in a row (maximum number of trials: 18). The learning score was calculated by attributing one point for each block correctly tapped until the criterion was reached; then it was added to the score corresponding to correct performance of the remaining trials (up to the 18th; maximum score: 144). Five minutes later, the DR part of the test was administered. The examiner asked Case 1 to reproduce the previously learned eight-block sequence. The score was the number of blocks correctly reproduced (maximum score: 8). She was tested individually in a quiet room with artificial lighting. She sat facing the examiner on a height-adjustable office chair in front of the CBT baseboard. Case 1’s performance on WM and the L and DR tests of the CBT was severely impaired when compared with the performances of the other women pilots and the women in the normative group (see Results section).

### Walking distance memory

To assess her ability to learn and remember spatial locations during navigation we used the Walking Corsi Test (WalCT: 16, 26). In the WalCT, she had to reproduce a walked sequence (previously demonstrated by the examiner) and to stop at different locations. The WalCT is a larger version of the CBT (3 × 2.5 m; scale 1:10 of the CBT), which is set up in an empty room. It consists of nine squares placed on a carpet in the same positions as in the standard CBT. The examiner shows the sequence by walking on the carpet and stopping on each square for 2 s. In this study, she had to repeat the exact sequence by walking and stopping on the squares included in the sequence. Also in the WalCT, she had to perform three different tasks: topographical working memory (TWM), in which a square span was obtained; topographical learning (TL), in which she had to learn an eight-square sequence following the same procedure and adopting the same learning criterion as in the CBT; and topographical delayed recall (TDR), in which Case 1 had to perform the eight-square sequence after five minutes had elapsed.

Results showed that Case 1’s TWM did not differ from that of controls (i.e., women pilots and nonpilots). Case 1’s performance on the TL and TDR after five minutes was comparable to that of controls (see Table 1 and Results section).

**Table 1:** **Case 1’s and average controls’ (i.e., pilots vs. nonpilots) performances in reaching and walking distance memory tests**

Test	Case 1 score pregnancy	Case 1 score 1 year postpartum	ItaF pilots	nonpilots
**Reaching distance memory**				
**CBT**				
Working memory	**5**	**5**	Mean = 5.8 S.D. = .42	Mean = 5.27 S.D. = .80
			Pre CH: t = -1.816; p = 0.05	Pre CH: t = -0.332; p = 0.74 n.s.
			Post CH: t = -1.816; p = 0.05	Post CH: t = -0.332; p = 0.74 n.s.
8-blocks sequence	**76**	115	Mean = 123.10 S.D. = 10.33	Mean = 123.34 S.D = 13.07
Learning			Pre CH: t = -4.347; p < .001	Pre CH: t = -3.561; p < .001
			Post CH: t = -0.748; p = .23 n.s.	Post CH: t = -0.627; p = .27 n.s.
Delayed recall	**6**	8	Mean = 7.8 S.D = 0.42	Mean = 6.93 S.D = 1.91
			Pre CH: t = -4.086; p < 0.01	Pre CH: t = -4.79; p = 0.32 n.s.
			Post CH: t = 0.454; p = 0.33 n.s.	Post CH: t = 0.551; p = 0.29 n.s.
**Walking distance memory test**				
**WalCT**				
Topographical working memory	7	7	Mean = 5.7 S.D = 1.42	Mean = 5.03 S.D = 1.15
			Pre CH: t = .873; p = .20 n.s.	Pre CH: t = 1.68; p = .05 n.s.
			Post CH: t = .873; p = .20 n.s.	Post CH: t = 1.68; p = .05 n.s.
8-squares sequence	142	138	Mean = 136.8 (S.D. = 4.16)	Mean = 131.31 (S.D. = 8.47)
Topographical learning			Pre CH: t = 1.192; p = .13 n.s.	Pre CH: t = 1.192; p = .13 n.s.
			Post CH: t = -0.275; p = .40 n.s.	Post CH: t = 0.77; p = .22 n.s.
Topographical delayed recall	8	8	Mean = 8 (S.D. = .05)	Mean = 7.72 (S.D. = 1.03)
			Pre CH: t = .00; p = .50 n.s.	Pre CH: t = .267; p = .40 n.s.
			Post CH: t = .00; p = .50 n.s.	Post CH: t = .267; p = .40 n.s.

Performances that were below the cut-off or insufficient are in bold.

**ItaF =** Italian Air Force; CBT = Corsi Block-Tapping Test; WalCT = Walking Corsi Test; S.D. = standard deviation; CH = Crawford & Howell’s 1998 analysis; n.s. = not significant.

## Results

The performance of Case 1 and controls (i.e., both women pilots and nonpilots) was compared using Crawford and Howell’s ([27]; CH) analysis with the computer program SINGLIMS.EXE. This analysis uses a modified t-test, described in Sokal and Rohlf [28], and is the most suitable one for estimating the abnormality of individual scores when the normative sample is small (i.e., less than 50 subjects).

We also compared Case 1’s performance on the CBT and the WalCT to determine whether there were any dissociations in her performance of the two tasks. This comparison was made using the computer program DISSOCS.EXE. [29]. It showed Case 1’s discrepancy from the control sample by estimating the percentage of the control population that exhibited a more extreme discrepancy than that of Case 1. We also analysed Case 1’s performance during pregnancy and postpartum to determine whether she was recovering and the magnitude of the recovery. To assess this aspect we used the C_CTC.EXE [30] program, which compared the scores of two of the two different moments, that is, during pregnancy and postpartum, as two separate events.

Results for each test are described below and shown in Table 1.

Crawford analysis (DISSOCS.EXE) showed a classical dissociation between Case 1’s CBT learning (z score = -4.56) and WalCT learning (z score = 1.25) scores. Compared with the performance of the women pilots’ group, Case 1’s performance showed a significant discrepancy (t = 4.44; p < .01). In fact, only 0.08% of the control group performed more discrepantly than Case 1. This incongruous performance was no longer present postpartum (CBT z score = -0.784; WalCT z score = 0.288; t = .858; p = .41 n.s.). The discrepancy was also present when Case 1’s performance was compared with that of the non-pilot women’s group (CBT z score = -3.622; WalCT z score = 1.262; t = 4.146 p < .001). When we compared her performance in the CBT during and one year after delivery, a significant difference emerged in Case 1’s ability to learn in reaching distance (t = -2.67; p < .01); however, there was no significant difference in her WalCT performance (t = .68; p = n.s.). In reaching space, she also failed in working memory (see Table 1), but this discrepancy did not reach significance when compared with the performance of the pilots’ group (CBT z score = -1.905; WalCT z score = .915; t = 2.03; p = 0.07 n.s.) and the non-pilots’ group (CBT z score = -0.338; WalCT z score = 1.713; t = 1.792; p = .08 n.s.).

## Discussion

Case 1 is an expert military pilot who showed dissociated performance in reaching versus topographical memory during pregnancy. To our knowledge this is the first investigation of specific aspects of visuo-spatial memory in a pregnant woman. She was evaluated during the second trimester of pregnancy and one year after delivery, when her hormonal values (estradiol and progesterone) had returned to normal. To investigate working memory and long-term memory she was submitted to the CBT and the WalCT on two different occasions. We compared her results during and after pregnancy with those of a group of pilots and a normative group matched for age, gender and education. At the first assessment (during pregnancy) she failed significantly in all aspects of reaching memory (working memory, learning and delayed recall). Surprisingly, all aspects of her topographical memory were preserved. This classical dissociation could be a specific effect of pregnancy or a protective effect due to her navigational expertise. However, Case 1’s poor performance during pregnancy, above all in reaching working memory, raises the issue of whether it was the pregnancy itself that impaired her performance or whether her baseline performance was simply worse than that of the controls. Indeed, we do not have a baseline of her performance before pregnancy and since her performance after pregnancy is not still completely recovered, even if improved, this could leave open the question if she could be worse than other women pilots also before her pregnancy. In any case, it is important to stress that a very strict selection is made at the beginning of the career in aviation that excludes people with low spatial abilities (including visuo-spatial working memory) and also rules out most women candidates. Indeed, last year the following data were published regarding candidates who attempted to enter the Aviation Academy: out of all women admitted to the selection only 66.67% passed the visuo-spatial test battery and only 16.67% of the remaining sample passed the mathematics tests. On the contrary, out of all men admitted to the selection 86.60% passed the visuo-spatial test battery and 44.04% of the remaining sample passed the mathematics tests. For this reason, we support the hypothesis that her low performance in the reaching memory task was a specific effect of hormonal changes during pregnancy and not an undiagnosed pre-pregnancy condition. Also her improvement after pregnancy in all other memory aspects suggests that she is coming back to her previous baseline. Indeed, she did not differ anymore from control groups in the postpartum period. It is also important to highlight that postpartum recovery in the general population is described as a broad spectrum ranging from two days to up to two months or longer [4, 31, 32] and no studies has still estimated the specific range in which these changes happened.

Sex differences in spatial abilities are not consistently found across studies because spatial ability encompasses three major dimensions: visualisation, orientation, and space relations [33]. Large differences that favour men are found in some visuo-spatial tasks, such as mental rotation, and spatiotemporal tasks [34]. Kimura and Hampson [35] reported that woman’s work performance changes in accordance with oestrogen fluctuations during their periods. In particular, when this hormone increases, spatial skills are minimized and manual and talking skills are maximized. Since visuo-spatial memory is also a multicomponential function, some components could be influenced more than others due to different hormonal levels in pregnancy and postpartum. In this case we could not exclude that a specific effect of navigational expertise preserved all aspects of topographical memory.

Caution should be taken in drawing conclusions about the absence of effects on topographical memory in pregnancy. Indeed, Piccardi *et al.* [16] found that men showed a larger span on the WalCT than on the CBT, whereas women’s spans did not differ on the two tests. Men were also faster than women in learning the supra-span sequence and in general were better in performing the WalCT than the CBT [16]. Women were slower in learning the path from a map and needed more repetitions to learn it in the real environment, but once they had learned the path there were no sex differences in delayed recall [36]. According to the hypothesis of Coluccia and Iosue [37] gender differences can be explained by the visuo-spatial WM load. Therefore, a WM failure during the typical hormonal changes of pregnancy could be in line with the difficulty of a control group of non-pregnant women in solving active working memory tasks. Indeed, sex differences are often reported in the CBT (see [38, 39]) that favour men with respect to women. Verde *et al.* [40] found that sex differences in mental rotation tasks were not present in a pilot population but that these differences were strong in a non-pilot population. In this study women pilots performed the same task as men pilots and as men non-pilots. During pregnancy, she performed similarly to the non-pilot women and performed even worse than this group in delayed recall. Although we were unable speculate about whether the general population of pregnant women would show a performance decline on the CBT, we can hypothesise that visuo-spatial working memory in reaching distance could have been impaired by the hormonal changes that occur during pregnancy. She did not show any deficits in the WalCT, thus showing that her topographical working memory was significantly larger than that of the non-pilot women. However, her walking memory performance could have been preserved due to a protective effect of her expertise in navigation regardless of her state. It has been widely demonstrated, however, that women prefer a route strategy based on egocentric memory for navigating (e.g., [41–43]). Recently, Nemmi *et al.* [19] performed an fMRI study comparing CBT and WalCT learning sequences and found that the retrosplenial cortex was involved in performance of the WalCT. These authors found that activation in the region in which the calcarine sulcus joins the parieto-occipital sulcus, which has been found active in several navigational tasks [44, 45]. Indeed, according to Byrne and Becker [46] this area might also transform egocentric representations into an allocentric frame and vice versa. This anatomical functioning evidence indicates why an egocentric strategy might be advantageous in performing the WalCT with respect to the CTB. In fact, in other studies women showed a dishomogeneous pattern in performing the CBT and the WalCT, which suggests that they use egocentric strategies in both types of memory.

Another interesting point is that in reaching distance the temporal storage of memory was recovered in a different way, showing that WM but not long-term memory is particularly sensitive to hormonal fluctuations. This evidence is in line with the current idea that most sex differences in solving spatial cognitive tasks are related to the active visuo-spatial working memory load.

The assessment of Case 1 (i.e. a woman with high spatial ability and without any complaints of depression, sleep disruption or fatigue) improved our knowledge of the memory changes that occur during pregnancy. Furthermore, the dissociation between her performance in reaching and walking distance memory tests is interesting because her expertise could have mitigated her low walking space memory performance. This can be explained as a factor protecting her experience in human navigation and preventing the complete failure of her visuo-spatial memory by saving the topographical component. Note that a military pilot is engaged in a range of visuo-spatial cognitive tasks that require different types of memory, which might be recovered in varying ways at different times postpartum. In the literature, postpartum recovery in the general population is described as a broad spectrum ranging from two days to up to two months or longer [4, 31, 32].

The above observations must be carefully considered when women pilots resume flight duties after delivery and suggest that a detailed neuropsychological assessment should be made to investigate different aspects of memory.

## Conclusions

The present case points out the importance of assessing spatial abilities that might be affected by hormonal changes during pregnancy and postpartum also in experts to guarantee their health and to determine when they should return from maternity leave.

## Consent

Written informed consent was obtained from the patient for publication of this Case Report. A copy of the written consent is available for review by the Editor-in-Chief of this journal.
